# Supplementary material for: End Sequence Analysis Toolkit (ESAT) expands the extractable information from single-cell RNA-seq data
Source: Genome Res. 2016 Oct;26(10):1397–410. doi: 10.1101/gr.207902.116 (PMC5052061; doi:10.1101/gr.207902.116)
Supplement: Supplemental Material [file supp_gr.207902.116_Supp_Materials.tar.gz › ESAT-esat_v0.1/doc/html/classbroad_1_1core_1_1annotation_1_1_repeat_masker_annotation.html]

ESAT: broad.core.annotation.RepeatMaskerAnnotation Class Reference


ESAT


- Main Page
- Related Pages
- Packages
- Classes
- Files

- Class List
- Class Index
- Class Hierarchy
- Class Members

All Classes Namespaces Files Functions Variables Enumerator Pages

- broad
- core
- annotation
- RepeatMaskerAnnotation

Public Member Functions |
List of all members

broad.core.annotation.RepeatMaskerAnnotation Class Reference

Inheritance diagram for broad.core.annotation.RepeatMaskerAnnotation:

[legend]

Collaboration diagram for broad.core.annotation.RepeatMaskerAnnotation:

[legend]

|  |  |
| --- | --- |
| Public Member Functions | |
|  | RepeatMaskerAnnotation () |
|  | |
|  | RepeatMaskerAnnotation (String name) |
|  | |
|  | RepeatMaskerAnnotation (GenomicAnnotation ga) |
|  | |
|  | RepeatMaskerAnnotation (String[] info) throws ParseException |
|  | |
|  | RepeatMaskerAnnotation (String chr, int start, int end, String strand, String repName, String repClass, String repFamily) |
|  | |
|  | RepeatMaskerAnnotation (int bin, int alignmentScore, int mismatches, int deleted, int inserted, String chr, int start, int end, int basesLeftAfterMatch, String strand, String repName, String repClass, String repFamily, int repStart, int repEnd, int repLeft) |
|  | |
| String | getRepeatName () |
|  | |
| String | getRepeatClass () |
|  | |
| String | getRepeatFamily () |
|  | |
| String | toString () |
|  | |
| Public Member Functions inherited from broad.core.annotation.BasicGenomicAnnotation | |
|  | BasicGenomicAnnotation (String name) |
|  | |
|  | BasicGenomicAnnotation (String name, String chr, int start, int end) |
|  | |
|  | BasicGenomicAnnotation (String name, String chr, int start, int end, Strand strand) |
|  | |
|  | BasicGenomicAnnotation (LightweightGenomicAnnotation ga) |
|  | |
|  | BasicGenomicAnnotation (Annotation ga) |
|  | |
|  | BasicGenomicAnnotation (GenomicAnnotation ga) |
|  | |
|  | BasicGenomicAnnotation (String[] info) |
|  | |
| int | getLength () |
|  | |
| String | toString () |
|  | |
| String | getId () |
|  | |
| void | setId (String id) |
|  | |
| void | reduceToDifference (GenomicAnnotation other) |
|  | |
| List< Annotation > | disect (Annotation a) |
|  | |
| List< Annotation > | disect (List<?extends Annotation > disectors) |
|  | |
| boolean | isFlankedBy (TwoSubjectAnnotation twoSubjectAnnotation, int buffer) |
|  | |
| int | lastIdxWhereListSearchBroke () |
|  | |
| Sequence | getSequence () |
|  | |
| void | setSequence (Sequence sequence) |
|  | |
| void | setOrientation (boolean orientation) |
|  | |
| void | setThreePrimeBuffer (int bufferSize) |
|  | |
| void | setFivePrimeBuffer (int bufferSize) |
|  | |
| int | getFivePrimeBases () |
|  | |
| int | getThreePrimeBases () |
|  | |
| int | compareTo (GenomicAnnotation arg0) |
|  | |
| int | getOrientedEnd () |
|  | |
| int | getOrientedStart () |
|  | |
| void | addBlock (String name, int start, int end) |
|  | |
| boolean | mayHaveBlocks () |
|  | |
| Public Member Functions inherited from broad.core.annotation.BasicLightweightAnnotation | |
|  | BasicLightweightAnnotation () |
|  | |
|  | BasicLightweightAnnotation (String chr, int start, int end) |
|  | |
|  | BasicLightweightAnnotation (String chr, int start, int end, String orientation) |
|  | |
|  | BasicLightweightAnnotation (String chr, int start, int end, Strand orientation, String name) |
|  | |
|  | BasicLightweightAnnotation (String chr, int start, int end, String orientation, double Scr) |
|  | |
|  | BasicLightweightAnnotation (String chr, String start, String end) |
|  | |
|  | BasicLightweightAnnotation (LightweightGenomicAnnotation annotation) |
|  | |
|  | BasicLightweightAnnotation (Annotation annotation) |
|  | |
| String | getChromosomeString () |
|  | |
| int | getDistanceTo (LightweightGenomicAnnotation other) |
|  | |
| double | getExtraScore (int i) |
|  | |
| List< Double > | getExtraScores () |
|  | |
| String | getLocationString () |
|  | |
| long | getMiddle () |
|  | |
| int | getOverlap (LightweightGenomicAnnotation other) |
|  | |
| double | getScore () |
|  | |
| void | setReversedOrientation (boolean isInReversedOrientation) |
|  | |
| void | setScore (double score) |
|  | |
| boolean | almostEqual (LightweightGenomicAnnotation other, int fudge) |
|  | |
| boolean | contains (LightweightGenomicAnnotation other) |
|  | |
| boolean | inReversedOrientation () |
|  | |
| boolean | overlaps (Collection<?extends Annotation > others, int buffer) |
|  | |
| boolean | overlaps (Collection<?extends Annotation > others) |
|  | |
| void | addExtraScore (double score) |
|  | |
| void | removeExtraScores () |
|  | |
| void | setBoundariesFromAnnoations (List<?extends GenomicAnnotation > annotations) |
|  | |
| void | stitchTo (LightweightGenomicAnnotation other) |
|  | |
| void | takeIntersection (LightweightGenomicAnnotation other) |
|  | |
| void | takeUnion (LightweightGenomicAnnotation other) |
|  | |
| LightweightGenomicAnnotation | intersect (LightweightGenomicAnnotation other) |
|  | |
| Collection<?extends Annotation > | intersect (Collection<?extends Annotation > annotations) |
|  | |
| int | hashCode () |
|  | |
| int | length () |
|  | |
| String | toUCSC () |
|  | |
| String | toString () |
|  | |
| int | compareTo (LightweightGenomicAnnotation b) |
|  | |
| void | setOrientation (String orientation) |
|  | |
| String | getChromosome () |
|  | |
| void | setChromosome (String chr) |
|  | |
| Public Member Functions inherited from broad.core.annotation.LightweightGenomicAnnotation | |
| int | getStart () |
|  | |
| int | getEnd () |
|  | |
| String | getName () |
|  | |
| void | setName (String name) |
|  | |
| void | setStart (int start) |
|  | |
| void | setEnd (int end) |
|  | |
| Public Member Functions inherited from umms.core.annotation.Annotation | |
| int | getSAMStart () |
|  | |
| int | getSAMEnd () |
|  | |
| String | getReferenceName () |
|  | |
| String | getChr () |
|  | |
| Strand | getStrand () |
|  | |
| boolean | hasOrientation () |
|  | |
| boolean | isNegativeStrand () |
|  | |
| int | numBlocks () |
|  | |
| List<?extends Annotation > | getBlocks (boolean oriented) |
|  | |
| int | getSize () |
|  | |
| int | size () |
|  | |
| int | getLengthOnReference () |
|  | |
| boolean | isUnoriented () |
|  | |
| int | getReferenceCoordinateAtPosition (int positionInAnnotation) |
|  | |
| int | getPositionAtReferenceCoordinate (int referenceCoordinate) |
|  | |
| int | getReferenceCoordinateAtPosition (int positionInAnnotation, boolean ignoreOrientation) |
|  | |
| int | getPositionAtReferenceCoordinate (int referenceCoordinate, boolean ignoreOrientation) |
|  | |
| void | setOrientation (char orientation) |
|  | |
| void | setOrientation (Strand orientation) |
|  | |
| void | setOrientedStart (int orientedStart) |
|  | |
| void | setOrientedEnd (int orientedEnd) |
|  | |
| void | setReferenceName (String refName) |
|  | |
| boolean | equals (Annotation other) |
|  | |
| void | expand (int deltaStart, int deltaEnd) |
|  | |
| Annotation | trim (int deltaStart, int deltaEnd) |
|  | |
| void | shift (int delta) |
|  | |
| void | moveToCoordinate (int coordinateInReference) |
|  | |
| Annotation | copy () |
|  | |
| Annotation | minus (Annotation other) |
|  | |
| Annotation | minus (Collection<?extends Annotation > others) |
|  | |
| int | getDistanceTo (Annotation other) |
|  | |
| String | toBED () |
|  | |
| String | toBED (int r, int g, int b) |
|  | |
| String | toShortBED () |
|  | |
| String | toBEDGraph () |
|  | |
| String | getFullInfoString () |
|  | |
| boolean | overlaps (Annotation other, int buffer) |
|  | |
| boolean | overlaps (Annotation other) |
|  | |
| boolean | overlaps (Annotation other, int buffer, boolean considerOrientation) |
|  | |
| boolean | overlaps (Annotation other, boolean considerOrientation) |
|  | |
| boolean | overlapsStranded (Annotation other) |
|  | |
| int | getOverlap (Annotation other) |
|  | |
| boolean | contains (Annotation other) |
|  | |
| Annotation | union (Annotation other) |
|  | |
| Annotation | intersect (Annotation other) |
|  | |
| List< Annotation > | intersect (List<?extends Annotation > annotations) |
|  | |
| int | compareToAnnotation (Annotation b) |
|  | |
| void | stitchTo (Annotation next) |
|  | |
| String | toCigar () |
|  | |
| boolean | fullyContains (Annotation annotation) |
|  | |
| Annotation | complement () |
|  | |
| boolean | equals (Annotation other, boolean useOrientation) |
|  | |
| Collection<?extends Annotation > | getSpliceConnections () |
|  | |
| int | getMidpoint () |
|  | |
| Public Member Functions inherited from broad.core.annotation.GenomicAnnotation | |
| void | setReversedOrientation (boolean isInReversedOrientation) |
|  | |
| void | setOrientation (String orientationString) |
|  | |
| Strand | getOrientation () |
|  | |
| String | getLocationString () |
|  | |
| List<?extends Annotation > | getBlocks () |
|  | |
| Public Member Functions inherited from broad.core.annotation.Feature | |
| String | getName () |
|  | |
| double | getScore () |
|  | |

|  |  |
| --- | --- |
| Additional Inherited Members | |
| Static Public Member Functions inherited from broad.core.annotation.BasicLightweightAnnotation | |
| static Collection  < BasicLightweightAnnotation > | mergeAllOverlappers (TreeSet< BasicLightweightAnnotation > regions) |
|  | |
| static LightweightGenomicAnnotation | createFromUCSC (String ucsc) |
|  | |
| static List< Annotation > | stitchList (Collection<?extends Annotation > sortedList, int maxDistanceToStitch) |
|  | |
| Protected Member Functions inherited from broad.core.annotation.BasicGenomicAnnotation | |
|  | BasicGenomicAnnotation () |
|  | |
| Protected Member Functions inherited from broad.core.annotation.BasicLightweightAnnotation | |
| void | setStart (String data) |
|  | |

## Constructor & Destructor Documentation

|  |  |  |  |  |
| --- | --- | --- | --- | --- |
| broad.core.annotation.RepeatMaskerAnnotation.RepeatMaskerAnnotation | ( |  | ) |  |

|  |  |  |  |  |  |
| --- | --- | --- | --- | --- | --- |
| broad.core.annotation.RepeatMaskerAnnotation.RepeatMaskerAnnotation | ( | String | *name* | ) |  |

|  |  |  |  |  |  |
| --- | --- | --- | --- | --- | --- |
| broad.core.annotation.RepeatMaskerAnnotation.RepeatMaskerAnnotation | ( | GenomicAnnotation | *ga* | ) |  |

Here is the call graph for this function:

|  |  |  |  |  |  |
| --- | --- | --- | --- | --- | --- |
| broad.core.annotation.RepeatMaskerAnnotation.RepeatMaskerAnnotation | ( | String[] | *info* | ) | throws ParseException |

Here is the call graph for this function:

|  |  |  |  |
| --- | --- | --- | --- |
| broad.core.annotation.RepeatMaskerAnnotation.RepeatMaskerAnnotation | ( | String | *chr*, |
|  |  | int | *start*, |
|  |  | int | *end*, |
|  |  | String | *strand*, |
|  |  | String | *repName*, |
|  |  | String | *repClass*, |
|  |  | String | *repFamily* |
|  | ) |  |  |

Here is the call graph for this function:

|  |  |  |  |
| --- | --- | --- | --- |
| broad.core.annotation.RepeatMaskerAnnotation.RepeatMaskerAnnotation | ( | int | *bin*, |
|  |  | int | *alignmentScore*, |
|  |  | int | *mismatches*, |
|  |  | int | *deleted*, |
|  |  | int | *inserted*, |
|  |  | String | *chr*, |
|  |  | int | *start*, |
|  |  | int | *end*, |
|  |  | int | *basesLeftAfterMatch*, |
|  |  | String | *strand*, |
|  |  | String | *repName*, |
|  |  | String | *repClass*, |
|  |  | String | *repFamily*, |
|  |  | int | *repStart*, |
|  |  | int | *repEnd*, |
|  |  | int | *repLeft* |
|  | ) |  |  |

Here is the call graph for this function:

## Member Function Documentation

|  |  |  |  |  |
| --- | --- | --- | --- | --- |
| String broad.core.annotation.RepeatMaskerAnnotation.getRepeatClass | ( |  | ) |  |

Here is the caller graph for this function:

|  |  |  |  |  |
| --- | --- | --- | --- | --- |
| String broad.core.annotation.RepeatMaskerAnnotation.getRepeatFamily | ( |  | ) |  |

Here is the caller graph for this function:

|  |  |  |  |  |
| --- | --- | --- | --- | --- |
| String broad.core.annotation.RepeatMaskerAnnotation.getRepeatName | ( |  | ) |  |

Here is the caller graph for this function:

|  |  |  |  |  |
| --- | --- | --- | --- | --- |
| String broad.core.annotation.RepeatMaskerAnnotation.toString | ( |  | ) |  |

Here is the call graph for this function:

---

The documentation for this class was generated from the following file:

- java/broad/core/annotation/RepeatMaskerAnnotation.java


---

Generated on Fri Aug 15 2014 15:10:51 for ESAT by  

 1.8.7
